# Associations of trait anger and the anger expression and regulation dimensions with food addiction in the LIFE-adult study

**DOI:** 10.1186/s40337-026-01802-8

**Published:** 2026-10-16

**Authors:** Felix S. Hussenoeder, Ines Conrad, Melanie Luppa, Margrit Löbner, Alexander Pabst, Christoph Engel, Nigar Reyes, Maryam Yahiaoui-Doktor, Heide Glaesmer, Andreas Hinz, Veronica Witte, Matthias L. Schroeter, Evelyn Medawar, Toralf Kirsten, Markus Loeffler, Arno Villringer, Steffi G. Riedel-Heller

**Affiliations:** 1 Institute of Social Medicine, Occupational Health and Public Health, Leipzig University, Ph.-Rosenthal-Str. 55, 04103 Leipzig, Germany; 2 LeiCeM - Leipzig Center of Metabolism, Leipzig University, Leipzig, Germany; 3 Institute for Medical Informatics, Statistics and Epidemiology (IMISE), Leipzig University, Leipzig, Germany; 4 Leipzig Research Centre for Civilization Diseases (LIFE), Leipzig University, Leipzig, Germany; 5 Department of Medical Psychology and Medical Sociology, Leipzig University, Leipzig, Germany; 6 Max-Planck-Institute for Human Cognitive and Brain Sciences, Leipzig, Germany; 7 Clinic for Cognitive Neurology, University Hospital Leipzig, Leipzig, Germany; 8 Department for Medical Data Science, University Medical Data Center Leipzig, Leipzig, Germany

**Keywords:** Anger, Anger Expression-In, Anger Expression-Out, Anger Control, Food Addiction, Mental Health, Personality, LIFE-Adult Study, Social Support

## Abstract

**Background:**

Food addiction (FA) has been linked to pathological eating, obesity, and a variety of detrimental mental health outcomes, yet the role of trait anger remains largely unexplored. This study examined whether trait anger and anger expression and regulation dimensions are associated with FA independently of the Big Five personality traits.

**Methods:**

We used data collected between 2011 and 2014 from 1,138 adults aged 20–79 years participating in the LIFE-Adult Study, a population-based German cohort study. Sequential Poisson regression analyses were conducted to examine associations of trait anger and the anger expression and regulation dimensions, i.e., anger expression-in, anger expression-out, anger control, with FA symptoms, adjusting for sociodemographic characteristics, social support, alcohol use, and the Big Five personality traits.

**Results:**

Higher trait anger was associated with higher FA symptom counts and remained significant after adjustment for the anger expression and regulation dimensions and sociodemographic characteristics. In the fully adjusted model, trait anger, lower social support, higher neuroticism, and lower conscientiousness were associated with higher symptom counts, whereas the anger expression and regulation dimensions were no longer significant.

**Conclusion:**

Our findings suggest that trait anger may be a relevant emotional correlate of FA beyond broader personality-related vulnerability, whereas specific anger expression and regulation dimensions appear to play a less central role. Given the cross-sectional design, conclusions regarding temporal ordering and causality remain limited.

## Abstract (plain language summary)

Some people experience eating in a way that feels hard to control, for example through strong cravings or repeated overeating despite negative consequences. This is sometimes described as food addiction. In this study, we examined whether people who generally tend to feel angry more often also report more symptoms of food addiction.

We used data from 1,138 adults from the LIFE-Adult Study in Leipzig, Germany. People with a stronger tendency to feel angry reported more symptoms of food addiction. This was still the case after taking into account factors such as age, gender, social support, alcohol use, and general personality traits. How people expressed or controlled their anger seemed less important than the overall tendency to feel angry.

The findings suggest that anger may be one emotional factor linked to difficult-to-control eating. Since the study measured anger and eating symptoms at one point in time, it cannot prove cause and effect. Still, the results indicate that a stable tendency to feel angry may be relevant for understanding why some people are more vulnerable to this type of eating.

## Introduction

Food addiction (FA) refers to an addiction-like pattern of eating, including symptoms such as loss of control, persistent craving, tolerance, and withdrawal in response to food consumption [1, 2]. Evidence suggests that FA and substance use disorders share risk factors, as well as neurobiological and hormonal correlates, personality features, and symptom profiles [3]. At the same time, FA remains a debated construct, particularly regarding its distinctiveness from established constructs of disordered eating [4]. FA has been associated with poor dietary choices, binge eating, bulimia nervosa, and obesity [5–7], and has therefore been proposed as a useful indicator of disordered eating in non-clinical populations [8, 9]. Compared with clinically diagnosed eating disorders, FA may be especially useful in subclinical samples because it captures meaningful variation in maladaptive eating in the general population. In addition, FA has also been associated with a broad range of mental health correlates, including anxiety, depression, personality disorders, psychotic disorders, chronic stress, quality of life, and self-esteem [10–13]. Most evidence in this area, however, is cross-sectional. One notable exception is a longitudinal study by Hussenoeder et al. [14], which identified bidirectional associations between anxiety and FA, underscoring the complexity of these relationships. Possible explanations include anxiety-related rumination and emotional eating contributing to FA, whereas overeating associated with FA may in turn affect physiological and neurobiological processes linked to anxiety.

Research on personality and FA has identified positive associations with impulsivity, harm avoidance, neuroticism, emotional dysregulation, urgency, lack of perseverance, and self-transcendence, as well as negative associations with self-directedness, cooperativeness, conscientiousness, and extraversion [15–18]. By contrast, the role of trait anger in FA remains largely unexplored. This is notable, given that anger has been linked to a wide range of maladaptive outcomes, including risky driving, abusive leadership, reactive aggressive behavior, violence, and suicidal behavior [19–22]. To the authors’ knowledge, the only exception is a recent study by Rahimnezhad et al. [23], which reported an association between FA and the single item “I often get angry about how others treat me.” The authors suggest that interpersonal conflict and the resulting negative affect may trigger compensatory eating. This is consistent with broader evidence linking anger to binge eating and suggesting that difficulties in regulating negative affect may contribute to pathological eating [24]. Trait anger may therefore be particularly relevant because it captures a stable tendency to experience anger and could represent an enduring vulnerability to FA and related pathological eating. It may also point to an anger-related pathway to FA that is not fully captured by broader personality traits or general distress. This question is also of practical relevance, given that anger is common in the general population. For example, in a nationally representative U.S. sample, the prevalence of inappropriate, intense, or poorly controlled anger was 7.8%, with higher rates among men and younger adults [25].

The present study therefore examined whether trait anger is associated with FA beyond the Big Five personality traits. Controlling for the Big Five allowed us to test whether any association with FA reflects a unique contribution of trait anger rather than broader dispositional personality differences. Since not only the experience of anger, but also the way it is expressed and regulated, may shape behavioral outcomes, we also examined anger-in, anger-out, and anger control. Beyond extending current knowledge on FA, these findings may also inform the prevention and treatment of FA and related eating pathology.

## Methods

### Study design

The Adult Study of the Leipzig Research Centre for Civilization Diseases (LIFE) is a population-based study conducted in Leipzig, Germany, through a collaboration of several clinical and epidemiological research groups. Between 2011 and 2014, adults aged 18 to 80 years were recruited via age- and sex-stratified random sampling from the local residents’ registry, with pregnancy as the sole exclusion criterion. Following written informed consent, participants completed a comprehensive assessment program including questionnaires, interviews, and medical examinations. Further information on the study design and assessment procedures can be found elsewhere [26, 27]. The LIFE-Adult Study included 10,000 participants at baseline. For the present analyses, the sample was restricted to participants who received the State–Trait Anger Expression Inventory, the NEO-FFI, and the Yale Food Addiction Scale (YFAS) (*n* = 1,209). After excluding cases with missing data on any study variable, the final analytic sample comprised 1,138 participants.

### Measures

#### Sociodemographic variables

Participants reported their age and gender during structured interviews carried out by trained study staff. In addition, information on educational attainment, employment status, and household income was collected to compute socioeconomic status [28]

#### Covariates

Social support was measured using the 5-item ENRICHD Social Support Instrument (ESSI; [29]; scale range: 1–5), with higher scores indicating greater perceived support. Cronbach’s α in the present sample was 0.91. Alcohol consumption over the past 12 months quantified in grams per day was assessed for beer, wine, and spirits, and these values were aggregated to estimate total consumption. Based on previously used sex-specific cut-offs for low-risk and excessive alcohol consumption (e.g., [30, 31]), alcohol intake was classified as low (female: ≤12 g/day; male: ≤24 g/day), medium (female: 13–40 g/day; male: 25–60 g/day), or high (female: >40 g/day; male: >60 g/day). The German version of the NEOFFI-30 [32, 33] was used to assess the Big Five personality traits—Neuroticism, Extraversion, Openness to Experience, Agreeableness, and Conscientiousness. The instrument comprises 30 items, with six items assessing each trait. After reverse coding where required, mean scores were calculated for each trait (range: 1–5), with higher scores indicating a stronger expression of the respective trait. Cronbach’s α values in the present sample were 0.83 for Neuroticism, 0.71 for Extraversion, 0.71 for Openness to Experience, 0.71 for Agreeableness, and 0.75 for Conscientiousness

#### Anger

Anger was assessed with the German version of the State-Trait Anger Expression Inventory (STAXI; [34]). For the present study, we used the scales for trait anger, i.e., the general tendency to experience anger, and the anger expression and regulation dimensions. The latter comprise anger held in (anger expression-in), anger directed outward (anger expression-out), and anger control (anger control). Items are rated on a four-point scale. Trait anger is calculated as the sum of 10 items (range: 10–40), whereas anger expression-in, anger expression-out, and anger control each comprise eight items (range: 8–32). Higher scores indicate a greater tendency to experience anger, hold anger in, express anger outward, or control anger, respectively. Examples of the content assessed include becoming angry easily (trait anger), keeping anger to oneself (anger expression-in), expressing anger toward other people or objects (anger expression-out), and trying to remain calm or restrain anger (anger control). Cronbach’s α values in the present sample were 0.82 for trait anger, 0.83 for anger expression-in, 0.80 for anger expression-out, and 0.82 for anger control

#### Food addiction

FA was measured with the Yale Food Addiction Scale (YFAS; 1, 2). The instrument comprises 25 items with dichotomous and Likert-type response options and covers seven symptom domains corresponding to DSM-IV substance dependence criteria, such as impaired control over eating and withdrawal. Subscale scores were derived using the algorithm described by Gearhardt et al. [1]. The total score reflected the number of FA symptoms and ranged from 0 to 7, with higher values indicating greater levels of FA symptoms

### Statistical analyses

Statistical analyses were performed using Stata 18.0 SE. Sequential Poisson regression models with robust standard errors were fitted to examine associations of trait anger, anger expression and regulation dimensions, sociodemographic characteristics, social support, alcohol consumption, and the Big Five personality traits with FA symptom counts.

## Results

Descriptive characteristics of the sample are shown in Table 1. The study included 1,138 participants aged 20–79 years, with a mean age of 54.1 years; 42.4% were women.

**Table 1:** General characteristics of the study population

Age (years)	Total Group (*N* = 1138)
	54.1 (16.9)
Gender (“female”)	483 (42.4%)
Socioeconomic status^1^	
Low	179 (15.7%)
Medium	675 (59.3%)
High	284 (25.0%)
Social support (ESSI, range: 1–5)	4.5 (0.7)
Alcohol consumption, g/day (female/male)^2^	
Low (≤ 12 g / ≤ 24 g)	868 (76.3%)
Medium (13–40 g/ 25–60 g)	220 (19.3%)
High (> 40 g/ > 60 g)	50 (4.4%)
Personality (NEO-FFI-30; range: 1–5)	
Neuroticism	2.4 (0.7)
Extraversion	3.4 (0.5)
Openness	3.4 (0.6)
Agreeableness	4.0 (0.6)
Conscientiousness	4.2 (0.5)
Anger (STAXI)	
Trait anger (range: 10–40)	17.2 (4.1)
Anger expression-in (range: 8–32)	16.2 (4.2)
Anger expression-out (range: 8–32)	11.4 (2.9)
Anger control (range: 8–32)	24.3 (4.2)
Food addiction (YFAS; range: 0–7)	1.4 (0.9)

Note. Continuous variables are presented as mean (SD), and categorical variables as n (%).

^1^Socioeconomic status was computed based on education, occupational status, and equivalent household income (28).

^2^Alcohol consumption categories were based on sex-specific average daily alcohol intake over the past 12 months.

The Poisson regression models are presented in Table 2. In Model 1, higher trait anger was associated with higher FA symptom counts. This association remained statistically significant after inclusion of anger expression and regulation dimensions in Model 2 and after additional adjustment for sociodemographic characteristics in Model 3. Anger expression-in was positively associated with FA symptom counts in Models 2 and 3, whereas anger expression-out and anger control were not associated with the outcome.

**Table 2:** Sequential Poisson regression models examining associations of anger-related variables and covariates with food addiction (*N* = 1,138)

Predictor	IRR	95% CI	*p*	IRR	95% CI	*p*	IRR	95% CI	*p*	IRR	95% CI	*p*
Trait anger	1.04	[1.026, 1.047]	< 0.001	1.02	[1.011, 1.039]	< 0.001	1.02	[1.011, 1.039]	< 0.001	1.02	[1.006, 1.034]	0.004
Anger expression-in	—	—	—	1.01	[1.002, 1.026]	0.022	1.01	[1.002, 1.026]	0.026	1.00	[0.992, 1.015]	0.534
Anger expression-out	—	—	—	1.01	[0.994, 1.028]	0.201	1.01	[0.995, 1.028]	0.170	1.01	[0.997, 1.029]	0.123
Anger control	—	—	—	1.00	[0.988, 1.007]	0.612	1.00	[0.992, 1.012]	0.736	1.01	[0.997, 1.017]	0.188
Age	—	—	—	—	—	—	1.00	[0.995, 0.999]	0.015	1.00	[0.996, 1.001]	0.132
Gender (ref.: female)	—	—	—	—	—	—	0.94	[0.876, 1.015]	0.119	0.96	[0.884, 1.038]	0.292
Socioeconomic Status(ref.: low)												
Medium	—	—	—	—	—	—	0.97	[0.875, 1.085]	0.635	1.00	[0.895, 1.110]	0.950
High	—	—	—	—	—	—	0.90	[0.807, 1.014]	0.085	0.95	[0.841, 1.070]	0.389
Social support	—	—	—	—	—	—	—	—	—	0.94	[0.886, 0.997]	0.039
Alcohol use (ref.: low)												
Moderate	—	—	—	—	—	—	—	—	—	1.00	[0.903, 1.098]	0.936
High	—	—	—	—	—	—	—	—	—	1.10	[0.927, 1.296]	0.285
Neuroticism	—	—	—	—	—	—	—	—	—	1.12	[1.039, 1.204]	0.003
Extraversion	—	—	—	—	—	—	—	—	—	1.02	[0.940, 1.097]	0.702
Openness	—	—	—	—	—	—	—	—	—	1.03	[0.973, 1.095]	0.286
Agreeableness	—	—	—	—	—	—	—	—	—	1.03	[0.954, 1.102]	0.495
Conscientiousness	—	—	—	—	—	—	—	—	—	0.89	[0.819, 0.976]	0.012
Wald χ² (df)	45.75 (1)			55.36 (4)			65.76 (8)			97.42 (16)		
Model p	< 0.001			< 0.001			< 0.001			< 0.001		
McFadden pseudo-R²	0.012			0.014			0.016			0.023		
AIC	2960.06			2960.45			2961.04			2958.11		
BIC	2970.13			2985.64			3006.37			3043.74		

Note. IRR = incidence rate ratio; CI = confidence interval; SES = socioeconomic status. Results are from Poisson regression models with robust standard errors. Dashes indicate that the predictor was not included in the respective model

In the fully adjusted model, trait anger remained positively associated with FA symptom counts. Lower social support, higher neuroticism, and lower conscientiousness were also associated with higher symptom counts, whereas all other predictors were not statistically significant. Model fit changed only modestly across the sequential models. Akaike Information Criterion (AIC) values differed only marginally, with the lowest value observed for Model 4, whereas Bayesian Information Criterion (BIC) favored the more parsimonious earlier models.

## Discussion

Our study provides evidence that trait anger is positively associated with FA symptoms, even after comprehensive adjustment for sociodemographic factors, social support, alcohol consumption, and the Big Five personality traits. The observed association persisted in the fully adjusted model, suggesting that trait anger captures variance in pathological eating behavior that is not reducible to broader personality dimensions. By contrast, the anger expression and regulation dimensions were not associated with FA symptoms in the fully adjusted model, which may indicate that trait anger is more relevant to FA than specific forms of anger expression and regulation. In addition, lower social support, higher neuroticism, and lower conscientiousness were associated with higher FA symptoms, consistent with the view that FA is related to a broader psychosocial vulnerability profile [16, 35, 36].

### Trait anger and FA

Consistent with prior research linking anger to a wide range of detrimental behaviors (e.g., 19, 20), our findings suggest that trait anger may play a distinct role in FA. Previous studies have associated FA with higher neuroticism and lower conscientiousness [16, 37], supporting the broader view that addictive-like eating behavior is partly embedded in trait-based deficits in self-regulation. While both higher neuroticism and lower conscientiousness were also associated with higher FA symptoms in the present study, trait anger remained significant when they were added to the regression. This suggests that anger is not merely a proxy for general emotional distress, but may represent a more specific pathway to pathological eating.

At the same time, the magnitude of the association between anger and FA was modest. This is not surprising in a large and diverse community-based sample, in which FA symptoms are likely to reflect the combined influence of multiple psychological, social, and behavioral factors rather than any single dominant determinant. Hence, small effects may still be meaningful, especially when the exposure is relatively common and stable over time, as is the case for personality traits [38]. Trait anger may therefore be best understood as an incremental correlate of FA symptoms.

One plausible explanation for our results is that individuals with high levels of trait anger may be more likely to engage in detrimental eating in response to perceived provocation, frustration, unfair treatment, social rejection, or anger rumination [39, 40]. This interpretation is consistent with prospective findings showing that hostility increased before and decreased after binge-only episodes in women with bulimia nervosa [41]. More broadly, negative affect and emotion dysregulation have been implicated in addictive-like eating [12, 42–44], suggesting that anger may constitute one specific emotional pathway within this larger pattern. In addition, literature has linked anger to impulsivity and disruptions in self control [45–47], which may increase susceptibility to impulsive, less inhibited forms of eating. In line with addiction models and with evidence for neurobiological and phenomenological overlap between FA and other addictive disorders [48–51], repeated pairing of anger states with rewarding food intake may reinforce problematic eating patterns over time.

### Anger expression and regulation

Anger expression and regulation dimensions showed limited and inconsistent associations with FA symptoms. Although anger expression-in was positively associated with higher FA symptom counts in partially adjusted models, this association did not persist after full adjustment. Anger expression-out and anger control were unrelated to FA across all models. Taken together, these findings suggest that trait anger may be more relevant to addictive-like eating than the specific ways in which anger is expressed or regulated.

At a theoretical level, this pattern appears somewhat at odds with models that emphasize maladaptive emotion regulation as a central driver of disordered eating and suggest that those eating behaviors may serve as a maladaptive strategy for managing aversive emotional states [52–54]. One possible interpretation is that trait anger captures a broader and more enduring vulnerability to dysregulated affective responding than anger expression-in, anger expression-out, and anger control. In addition, anger expression and regulation dimensions may matter primarily in specific affective or environmental contexts not captured here, such as acute stress or interpersonal conflict, a possibility that should be tested in future studies.

### Limitations

Several limitations warrant consideration. The cross-sectional design does not allow conclusions about temporal ordering or causality. The exclusion of participants with missing data may have introduced some selection bias. As expected in a population-based sample, the average FA symptom count was relatively low, which may limit the generalizability of our findings to individuals with more severe FA symptomatology. In addition, anger was assessed with the German version of the original STAXI rather than the revised STAXI-2, and FA symptoms were assessed using the original YFAS rather than the YFAS 2.0, which may somewhat limit comparability with more recent work. These limitations are balanced by important strengths, including the differentiated assessment of anger-related constructs, the large sample, and adjustment for broad personality traits.

### Implications for theory and practice

Our findings have potential implications for both theory and practice. At a theoretical level, they suggest that trait anger may deserve greater consideration in models of FA, which have largely focused on anxiety, depression, and broader forms of negative affect or emotion dysregulation [55–57]. More specifically, the present results raise the possibility that anger may represent a specific affective pathway to FA, beyond general distress and broad personality-related vulnerability.

From a public health perspective, trait anger may represent an overlooked marker of vulnerability, with potential relevance for early identification of individuals at elevated risk for pathological eating and prevention. Psychosocial interventions, particularly cognitive behavioral approaches, have shown beneficial effects on anger [58], suggesting that anger-related processes may represent a potential intervention target. Interventions may also directly address problematic eating, with evidence that psychological interventions can reduce emotional eating [59], while adapted motivational interviewing combined with cognitive behavioral therapy has been shown to reduce FA symptoms [60]. However, longitudinal and intervention studies are needed before implications for screening or treatment can be established.

## Data Availability

The dataset analysed during the current study are available from the corresponding author on reasonable request.
